# Advantages and Disadvantages of Breast Augmentation: Surgical Techniques, Outcomes and Future Directions

**DOI:** 10.7759/cureus.69846

**Published:** 2024-09-21

**Authors:** Christopher R Meretsky, Erik M Knecht, Anthony T Schiuma

**Affiliations:** 1 Surgery, St. George's University School of Medicine, Great River, USA; 2 Surgery, Chicago Medical School at Rosalind Franklin University, Illinois, USA; 3 Orthopedic Surgery, Holy Cross Hospital, Fort Lauderdale, USA

**Keywords:** breast augmentation, complications, patient satisfaction, postoperative pain, surgical techniques

## Abstract

Breast augmentation remains a highly sought-after cosmetic surgery, with various techniques available to enhance breast size using implants or fat transfer. This systematic review, spanning studies from 2003 to 2024 and adhering to PRISMA guidelines, evaluates the outcomes of different surgical approaches concerning scar quality, postoperative pain, patient satisfaction, and complications. The periareolar technique emerges as a favorable option, offering minimal postoperative pain, high patient satisfaction, and precise surgical control with subtle scarring. Transumbilical breast augmentation (TUBA) is noted for its scar-free approach, resulting in low pain and high satisfaction, though it requires specialized training and cannot use prefilled implants. The review highlights the varying complication rates across techniques: modern silicone implants, while safer than earlier injectable materials, still face risks such as rupture and capsular contracture. Autologous fat grafting is generally safer but can lead to issues like erythema and abscesses. The use of effective acellular dermal matrix (ADM) is linked to higher rates of seroma and infection compared to non-ADM procedures, although sterile human ADM shows a lower risk profile. The study underscores the importance of personalized surgical planning to optimize outcomes, as each technique offers distinct benefits and challenges. The findings suggest that future research should aim to refine these techniques and address their associated complications to further enhance patient satisfaction and surgical outcomes in breast augmentation.

## Introduction and background

Breast augmentation is a surgical procedure aimed at increasing breast size through implants or, less frequently, fat transfer. In the United States of America (U.S.), it is a highly popular cosmetic surgery. The Food and Drug Administration (FDA) restricted silicone-filled implants for cosmetic use in 1992 due to safety concerns, though they remained available for reconstructive purposes post-mastectomy. Extensive research eventually disproved the health risks associated with silicone implants, leading the FDA to lift the ban in 2006. By 2017, most breast augmentations used cohesive gel silicone implants, which are recommended for Magnetic Resonance Imaging (MRI) screening every two years to detect potential leaks. Despite this, adherence to screening is inconsistent, and MRI scans can produce false positives. Modern implants feature increasingly cohesive gels, enhancing firmness and potentially reducing rupture rates, though all implants are likely to eventually rupture over time [1-3]. Breast implants, available as saline-filled or silicone-filled, both have an outer silicone shell. Saline implants are typically inserted empty and filled with sterile saline during surgery, allowing adjustable volume. Silicone implants contain a viscous silicone gel and provide a softer, more natural feel, particularly beneficial for patients with thin soft tissue coverage. Implants can have a smooth or textured shell, with texturing used to reduce rotation in shaped implants and potentially lower capsular contracture rates in the sub-glandular pocket. Placement options include above (sub-glandular) or below (submuscular) the pectoralis muscle, with the choice depending on patient anatomy and surgeon preference. Common incision sites are the inframammary crease, trans-axillary, and peri-areolar areas. Breast augmentation, typically an outpatient procedure under general anesthesia, takes 45 to 90 minutes, with a recovery period of about a week for light activities and up to six weeks for full recovery [4].

One of the major problems related to breast augmentation is obtaining enough soft tissue cover; it is especially difficult in thin patients with severe secondary hypomastia. Lack of breast tissue and/or subcutaneous tissue can result in unfavorable aesthetic results such as implant visibility, implant edge validation, and shell ripple despite the achievement of increased volume and/or projection [5,6]. In response to this, several methods have been used to help camouflage the implants in patients with low cheekbones. Some of these are adjusting implant position to the subpectoral or dual-plane pocket technique [7] in the hope of achieving additional soft tissue coverage from the pectoralis major muscle. Moreover, the application of highly cohesive form-stable anatomic implants further minimizes the prospects of wrinkle and pique formation [8]. Other biomaterials, such as acellular dermal matrix, have also been employed in increasing soft tissue coverage and in handling issues relating to the soft tissue flap [9]. The introduction of silicone gel implants in 1962 marked the beginning of modern breast augmentation. Following a 1992 moratorium on silicone implants in the U.S., saline implants dominated North America, while silicone continued to be used globally. Cohesive gel implants became available in the U.S. in 2001 under an Investigational Device Exemption Study. Three companies, Sientra, Allergan, and Mentor, received FDA approval for fifth-generation implants, which are distinguished by features such as shell texture, implant dimensions, gel-shell interaction, gel fill ratio, degree of cross-linking, and form stability, reflecting significant advancements in implant technology [10].

Primary fat grafting during breast augmentation has gained popularity as a technique to enhance breast shape naturally and better conceal the underlying implants while increasing breast size. This approach, known as composite breast augmentation, involves combining prosthetic implants with autologous fat to manage both the core volume and the overlying soft tissue of the breast [5]. By using implants for volume and autologous fat for shaping, surgeons achieve greater precision in addressing breast asymmetries and refining breast contours [11]. This method overcomes some limitations of implant-only augmentation, particularly in patients with insufficient soft tissue coverage, allowing for a more natural and aesthetically pleasing result [6].

In breast augmentation, four types of incisions are used: transaxillary, inframammary, periareolar, and transumbilical. The inframammary incision offers full visual access for precise implant placement but leaves a visible scar. The periareolar incision heals inconspicuously but may affect nipple sensitivity and leave a scar. The transaxillary incision avoids breast scarring and suits saline and gel implants but may require a second incision for corrections. Transumbilical augmentation uses a hidden incision but is limited to saline implants and poses challenges in achieving proper hemostasis. Each option has specific risks and benefits, requiring careful consideration by the patient and surgeon [12].

Breast implants carry several risks and complications, including breast pain, changes in nipple and breast sensation, and the need for additional surgeries, potentially with implant removal. Complications also include capsular contracture, where scar tissue squeezes the implant and risks of rupture and deflation. Serious concerns involve breast implant-associated anaplastic large cell lymphoma (BIA-ALCL), other lymphomas, squamous cell carcinoma, and mesenchymal tumors. Additionally, implants may be linked to connective tissue diseases, reproductive issues, and systemic symptoms and can impact breastfeeding and children's health. These risks underscore the importance of informed decision-making for individuals considering breast implants [13]. Women undergoing breast augmentation frequently experience local complications, which are less frequent among patients receiving for cosmetic reasons than among those receiving postmastectomy on account of cancer or for cancer prophylaxis [14]. There has long been discussion about the best approach for breast augmentation, with many different factors fitting the needs of our patients' diverse morphologies [15].

This study aims to provide a comprehensive evaluation of various breast augmentation techniques, focusing on their impact on scar quality, postoperative pain, patient satisfaction, and complications. By systematically comparing the outcomes associated with different surgical methods, the study seeks to offer valuable insights that can guide surgeons in selecting the most appropriate approach tailored to individual patient needs, ultimately enhancing overall patient outcomes and satisfaction.

## Review

Methods

This systematic review was conducted following the Preferred Reporting Items for Systematic Reviews and Meta-Analyses (PRISMA) guidelines. Authors Christopher Meretsky, Eric Knecht, and Anthony Schiuma independently evaluated all eligible studies, resolving any disagreements through consensus. The study systematically reviews existing literature, clinical trials, and meta-analyses to assess the effectiveness of traditional and modern techniques in reducing scarring. A comprehensive literature search was performed using databases such as PubMed, Google Scholar, and the Cochrane Library, utilizing keywords including “SUB-PECTORAL BREAST AUGMENTATION SCAR QUALITY AND PATIENT SATISFACTION," “PRE-PECTORAL BREAST AUGMENTATION," “TRANSAXILLARY BREAST AUGMENTATION," “PERI-AREOLAR BREAST AUGMENTATION,” and “TRANSUMBILICAL BREAST AUGMENTATION." All the articles, systemic reviews, and meta-analyses that were published during the period 2003-2024 were included in the review. Inclusion criteria included studies that involved breast augmentation procedures, reporting scar quality, postoperative pain, and patient satisfaction.

**Figure 1:**
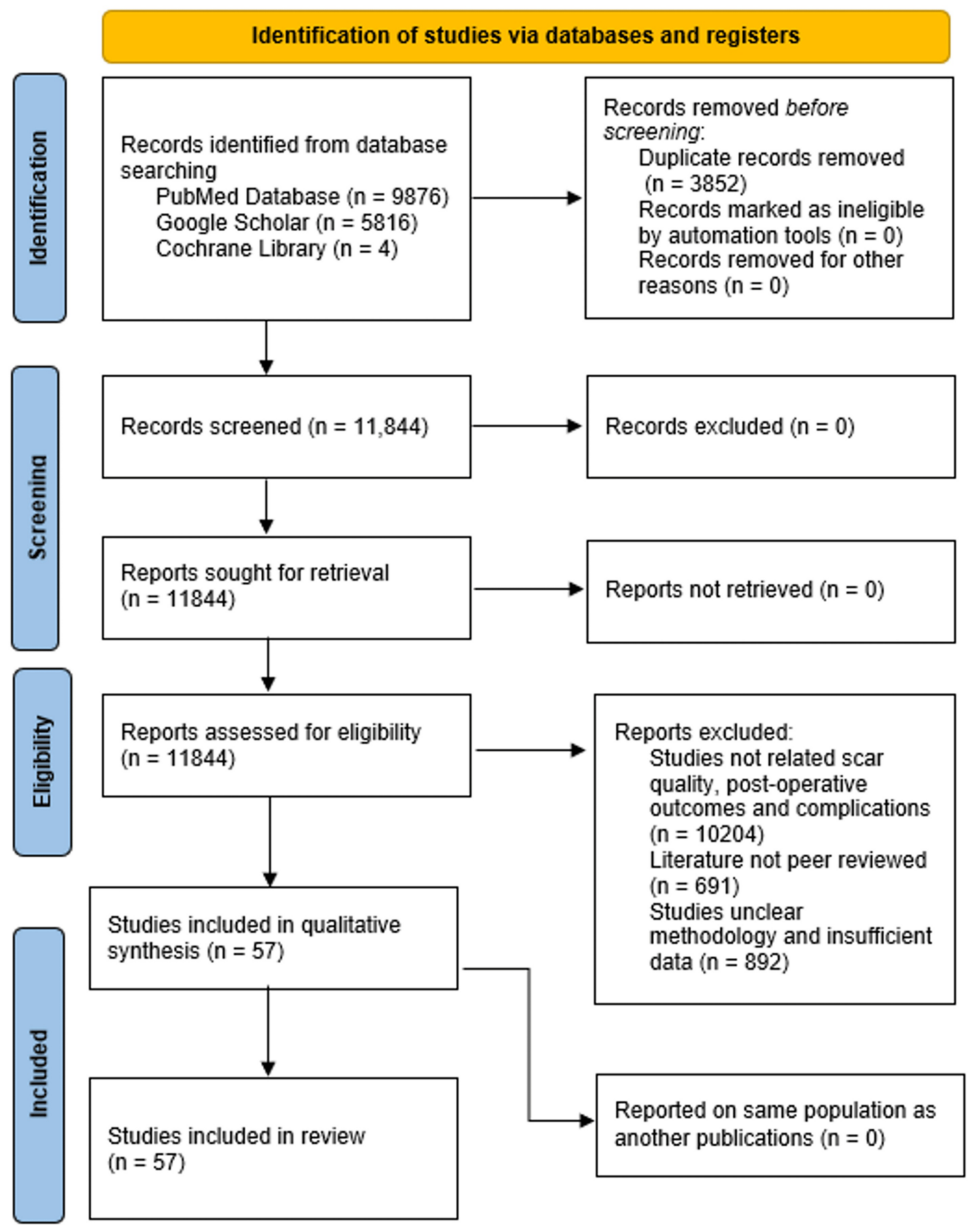
PRISMA flowchart: literature search and study selection n: number; PRISMA: Preferred reporting items for systematic reviews and meta-analyses

Figure 1 depicts a PRISMA (Preferred Reporting Items for Systematic Reviews and Meta-Analyses) flow diagram used to outline the study selection process in a systematic review. The process begins with the identification of studies, where 15,796 records were sourced from PubMed (9,876), Google Scholar (5,816), and the Cochrane Library (4). After removing 3,852 duplicate records, 11,844 unique records remained for screening. None of these records were excluded or could not be retrieved, leading to 11,844 reports being assessed for eligibility. During the eligibility assessment, 10,204 studies were excluded for not addressing relevant topics like scar quality, postoperative outcomes, and complications. An additional 691 studies were excluded due to lack of peer review, and 892 were excluded for unclear methodology or insufficient data. Ultimately, 57 studies met the criteria and were included in the qualitative synthesis and review. The diagram provides a clear and systematic overview of the study selection process.

Results

Procedure Types

Inframammary fold or sub-pectoral vs. prepectoral techniques: The inframammary fold (IMF) incision remains the most practiced approach in breast augmentation surgery, favored by plastic surgeons despite its primary drawback, the visibility of the scar. The popularity of this technique is largely attributed to its multiple advantages. It is relatively easy to learn and provides direct access to the submuscular, subpectoral, or subglandular planes. The IMF approach allows for precise dissection within the desired pocket boundaries without the need for an access tract between the entry site and the pocket, as required in other techniques. Additionally, it does not require expensive surgical equipment like endoscopes and accommodates the use of dependent drains should the surgeon's technique necessitate them [16]. The IMF incision is particularly preferred for patients with a well-developed inframammary crease that effectively conceals the scar. Proper placement of the incision within the new IMF is crucial, as the level of the inframammary crease is dynamic and typically descends post-augmentation. Ensuring accurate localization of the new IMF is, therefore, a cornerstone of successful breast augmentation, making the IMF incision a reliable and widely used approach [17,18]. Subpectoral Augmentation is a traditional technique where implants are placed beneath the pectoralis major muscle. This method often results in animation deformity, where muscle contractions cause the implant to move, and can lead to increased postoperative pain due to muscle involvement. Subpectoral augmentation may also cause more upper breast implant displacement and can affect the natural breast shape. The pre-pectoral augmentation technique involves placing the breast implants above the pectoralis major muscle, directly under the skin, and overlying the acellular dermal matrix (ADM) or synthetic mesh. The introduction of ADM has been crucial, as it reinforces the skin flaps, helping to maintain the integrity of the implant placement and reducing complications such as implant exposure. Prepectoral augmentation is associated with reduced postoperative pain, elimination of animation deformity (AD), and a more natural breast shape. It is particularly beneficial for patients with minimal or no breast ptosis. Recent advancements, including the use of ADM and improved surgical techniques, have made prepectoral augmentation a safer and more favorable option for many patients [19]. Table 1 summarizes the various studies on sub-pectoral vs. pre-pectoral techniques identified in this study.

**Table 1:** Summary of studies on sub-pectoral vs. pre-pectoral breast augmentation techniques IMF: Inframammary fold; AK: Akademikliniken; DTI: Direct-to-implant; BMI: Body mass index [20-27]

Study	Technique	Scar Quality	Postoperative Pain	Patient Satisfaction	Complication
Aboelatta, Yasser Abdallah et al. [20]	IMF Incision (Prepectoral)	90.6% excellent scar placement; no pruritic or pigmented scars	Effective management; no significant pain issues reported	High; all patients satisfied with results	Capsular contracture in 3 patients (350-400 mL implants)
Montemurro P et al. [21]	Short IMF Incision (AK method)	High scar quality (POSAS: 6.82 right, 6.51 left; VSS: 0.26 right, 0.10 left; MSS: 5.42 right, 5.23 left)	Not specifically assessed	High satisfaction (BREAST-Q: 83.86)	Minimal complications (5.1% minor wound issues, resolved within 2 weeks)
Walker NJ et al. [22]	Prepectoral vs. Subpectoral	Not specifically measured	Not specifically assessed	Subpectoral generally preferred aesthetically	Prepectoral: Higher implant exposure in BMI >35 kg/m2
Franceschini G et al. [23]	Prepectoral vs. Subpectoral	Not directly measured	Lower chronic pain in Prepectoral	Higher in Prepectoral (better skin sensibility)	PP: Shorter operative time; more cost-effective
Thangarajah F et al. [24]	Prepectoral vs. Subpectoral	No significant differences	No significant differences	No significant differences in satisfaction	Subpectoral: Higher major complications (1.41 vs. 0.47)
Manrique OJ et al. [25]	Prepectoral vs. Subpectoral (DTI)	Not explicitly measured	Not specifically assessed	High in both techniques; no significant differences	Comparable overall complications (Prepectoral: 7.2% vs. Subpectoral: 11.6%)
Bernini M et al. [26]	Prepectoral vs. Subpectoral	91% excellent in Prepectoral vs. 65% in Subpectoral	Less pain in Prepectoral due to no muscle manipulation	Higher in Prepectoral (BREAST-Q scores favored)	Prepectoral: No high-grade capsular contracture vs. 12% in Subpectoral
Catellani L et al. [27]	Prepectoral vs. Subpectoral	Superior in Prepectoral (81.25% symmetry)	Less pain in Prepectoral (lower analgesic consumption)	Higher in Prepectoral (better psychosocial well-being)	Prepectoral: Faster return to work (34.56 days vs. 57.31 days)

In a study by Aboelatta, Yasser Abdallah et al. [20], complications were relatively low, with capsular contraction being the most significant issue, reported in three patients who received implants of 350 to 400 mL. These cases were managed with capsulectomy and adjustments to the implant pocket or implant size. Postoperative seromas were observed in two patients from Group 2, which required implant removal after failed evacuation attempts. There were no recorded cases of postoperative hematoma, wound infection, or wound dehiscence. Additionally, hypertrophic scars developed in two patients, who were effectively treated with silicone sheets and intralesional steroids, eliminating the need for surgical intervention. Regarding the quality of scars, most patients (90.6%) had excellent scar placement, particularly within the new inframammary fold (IMF). Scars were well-concealed, especially in patients with a well-developed IMF. No painful, pruritic, or pigmented scars were reported. The quality of postoperative pain was not explicitly detailed, but the lack of reports on significant pain issues suggests effective management and overall patient comfort. Patient satisfaction was high, with all patients expressing satisfaction with their results. The study's marking technique for IMF incision placement was successful in achieving aesthetically pleasing outcomes, with scars well-hidden and minimal complications, contributing to high postoperative satisfaction [20].

Similarly, a study by Montemurro P et al. [21] reviewed 78 consecutive female patients who underwent primary breast augmentation using the Akademikliniken (AK) method and a short inframammary fold (IMF) incision. The results demonstrated high scar quality and overall patient satisfaction with minimal complications. Scar quality was assessed using the Patient and Observer Scar Assessment Scale (POSAS), Vancouver Scar Scale (VSS), and Manchester Scar Scale (MSS). The mean POSAS scores were low, with 6.82 for the right breast and 6.51 for the left breast, indicating favorable outcomes. VSS and MSS scores were similarly low, with VSS averaging 0.26 for the right breast and 0.10 for the left, and MSS scores averaging 5.42 and 5.23, respectively. Postoperative satisfaction was measured using the BREAST-Q “satisfaction with breasts” subscale, with a high mean score of 83.86 (out of 100), indicating very good patient satisfaction. The complication rate was minimal, with only 5.1% of patients experiencing minor superficial wound healing issues, all resolved conservatively within two weeks. The study concluded that a shorter IMF scar offers a competitive advantage by providing high scar quality and overall satisfaction without increasing complications, making it an attractive option in the social media-driven patient market.

A study by Walker NJ et al. [22], compared complication rates and outcomes between subpectoral and prepectoral implant-based breast reconstruction across different BMI groups. Among patients with a BMI over 35 kg/m2, prepectoral reconstruction had a higher rate of implant exposure, while those with a BMI over 25 kg/m2 experienced more minor asymmetry with the prepectoral technique. Although the odds of reoperation increased with BMI, this trend did not reach statistical significance. Aesthetic outcomes, rated through surveys, indicated that subpectoral reconstructions were generally preferred, particularly by physicians. Patients with a BMI under 25 kg/m2 reported better aesthetic satisfaction compared to higher BMI groups. Overall, while prepectoral reconstruction showed a trend toward higher complications with increasing BMI, both techniques demonstrated comparable clinical and cosmetic results, with subpectoral reconstruction being rated more favorably in terms of aesthetics [22].

Similarly, another study by Franceschini G et al. [23] compared outcomes of immediate prosthetic breast reconstruction (IPBR) using submuscular (SM) versus pre-pectoral (PP) placement of micro-polyurethane-foam-coated implants following nipple-sparing mastectomy (NSM). Among the 177 patients, the PP group had a shorter operative time of 70 minutes and demonstrated significant advantages in several areas. Specifically, patients in the PP group reported lower levels of chronic postoperative pain, less shoulder dysfunction, and better skin sensibility compared to the SM group. Aesthetic outcomes were also rated more favorably in the PP group, with trends suggesting better satisfaction in sports activity and sexual/relationship life. While scar quality was not directly measured, the overall postoperative satisfaction was higher in the PP group. Additionally, the PP technique was found to be more cost-effective. The findings suggest that PP-IPBR is a safe, effective, and economically advantageous option with superior postoperative outcomes compared to SM-IPBR [23].

Additionally, a retrospective study by Thangarajah F et al. [24] compared the outcomes of prepectoral and subpectoral immediate implant-based breast reconstruction (IBR) following skin- or nipple-sparing mastectomy in a cohort of 63 patients. Among these, 34 patients underwent prepectoral IBR, and 29 underwent subpectoral IBR. The analysis revealed that the subpectoral group experienced a significantly higher mean number of major complications per patient (1.41 vs. 0.47; p < 0.05), with a notably increased incidence of implant dislocation (p < 0.05). However, there were no significant differences in postoperative pain, scar quality, or patient satisfaction between the two groups, as measured by the Breast-Q questionnaire. The mean volume of implants used was nearly identical between the groups (292 ml for subpectoral vs. 293 ml for prepectoral; p = 0.975). Additionally, while the surgical procedure was longer in the subpectoral group (104 ± 28 vs. 80 ± 91 minutes; p < 0.05), hospital stay duration did not differ significantly (p = 0.111). Overall, despite higher complication rates in the subpectoral group, these did not appear to affect postoperative satisfaction or quality of life, underscoring the importance of considering both options based on individual patient needs [24].

Interestingly, a study by Manrique OJ et al. [25] compared single-stage direct-to-implant (DTI) breast reconstruction outcomes between prepectoral and subpectoral placements. Both techniques yielded similar results in terms of scar quality, postoperative pain, and satisfaction. Scar quality was not explicitly measured, but overall complications were comparable: 7.2% in the prepectoral group versus 11.6% in the subpectoral group, with no significant difference (p = 0.227). Postoperative pain was not specifically assessed but can be inferred from the similar complication rates. Patient-reported outcomes via BREAST-Q showed high satisfaction with both techniques, with mean scores of 75 for prepectoral and 73.9 for subpectoral, indicating no significant difference in overall satisfaction (p = 0.211). The study concludes that prepectoral DTI is a safe, reliable, and effective option with outcomes comparable to subpectoral DTI, particularly for patients with minimal or no breast ptosis who wish to maintain their breast size [25].

Subsequently, another study by Bernini M et al. [26] compared subcutaneous (prepectoral) and retropectoral (submuscular) breast reconstruction techniques with a median follow-up of 25 months. The subcutaneous group (G-2) showed superior scar quality, with 91% of cases rated as having an excellent aesthetic outcome compared to 65% in the retropectoral group (G-1). Despite better overall results, 9% of G-2 cases required fat grafting to correct visible implant borders and rippling. The subcutaneous technique resulted in less postoperative pain, contributing to higher patient comfort. The absence of muscle manipulation in this approach likely explains the reduced pain compared to the retropectoral method. Patient satisfaction was significantly higher in the subcutaneous group, with BREAST-Q scores favoring this approach. Specifically, 12% of G-1 cases required implant changes for functional or aesthetic reasons, whereas 0% of G-2 cases needed such revisions. Additionally, the capsular contracture rate was 12% (III-IV grade) in G-1, while G-2 had no instances of high-grade capsular contracture. These results suggest that the prepectoral approach offers better scar quality, reduced postoperative pain, and higher patient satisfaction, making it a viable alternative to the sub-pectoral technique, especially when skin flap viability and wound healing are favorable [26].

Similarly, a prospective nonrandomized study by Catellani L et al. [27] compared prepectoral breast reconstruction (PPBR) using acellular dermal matrix-wrapped implants with subpectoral reconstruction in 86 mastectomy patients. The study focused on scar quality, postoperative pain, and patient satisfaction. PPBR demonstrated superior aesthetic outcomes, with 81.25% of patients achieving satisfactory symmetry compared to just 10.81% in the subpectoral group (p < .001). This reduced the need for additional contralateral surgeries for symmetrization in PPBR patients (18.75% vs. 89.19%). Patients in the PPBR group experienced significantly less pain, as reflected by lower analgesic consumption on postoperative day (POD) 1 (p = .012) and POD 7 (p < .001). Additionally, the Brief Pain Inventory-Short Form (BPI-sf) results also showed lower pain scores for PPBR patients on the same PODs (p < .001). The BREAST-Q questionnaire revealed significantly higher satisfaction in the PPBR group, with better psychosocial well-being and aesthetic outcomes (p < .001). Furthermore, patients in the PPBR group returned to work sooner, averaging 34.56 days compared to 57.31 days for the subpectoral group (p < .001). Overall, the study found that PPBR provided better scar quality, reduced postoperative pain, and higher patient satisfaction, making it a favorable alternative to subpectoral reconstruction [27].

Transaxillary breast augmentation technique: The transaxillary approach for breast augmentation, first introduced in the 1970s [28], has undergone significant evolution, particularly with the integration of endoscopic technology. Initially, this method, while innovative, faced challenges such as potential hematoma, trauma, and difficulties in creating a defined inframammary crease due to its blind technique. Early endoscopic adaptations improved the procedure by reducing bleeding and allowing for more precise dissection, yet issues like tissue damage and incomplete muscle dissection persisted. Modern advancements have transformed the transaxillary approach into a refined technique involving sharp electrocautery dissection under direct endoscopic vision [29,30]. This approach ensures a bloodless pocket, minimized tissue trauma, and complete division of the costal origin of the pectoralis major muscle, leading to well-defined and symmetrical inframammary creases. The result is a technique that now offers comparable outcomes to traditional inframammary approaches, with benefits including quicker recovery, reduced pain, and less implant displacement [31]. Table 2 outlines the summary of studies included in transaxillary breast augmentation technique.

**Table 2:** Summary of studies on transaxillary breast augmentation approach TBA: Transaxillary breast augmentation [31-39]

Study	Techniques	Scar Quality	Postoperative Pain	Complications	Patient Satisfaction	Key Findings
Ana Claudia Weck Roxo [32]	Endoscopic vs. Non-Endoscopic Transaxillary	High satisfaction in both groups	Slightly higher in non-endoscopic group	Minimal complications in both groups	Comparable between groups	Endoscopic assistance prolongs operative time but does not significantly improve outcomes or reduce complications.
Philippe A. Giordano et al. [33]	Endoscopic Transaxillary	Hidden scars, no concerns reported	Minimized due to avoidance of muscle dissection	Low complication rate (6.5%), low reoperation rate (6.2%)	High overall satisfaction	The approach effectively addresses minor to moderate breast deformities while preserving aesthetic outcomes.
Sim HB [31]	Endoscopic Transaxillary with Sharp Electrocautery	No issues with scar quality, particularly beneficial for East Asian women	Reduced due to minimized tissue damage	Capsular contracture in 2.16% of patients	High patient satisfaction, quick recovery	Effective in avoiding visible scars, reducing pain, and achieving high patient satisfaction with low complication rates.
Momeni A et al. [34]	Inframammary vs. Endoscopic Transaxillary	Higher satisfaction with transaxillary approach due to hidden scar	Similar between approaches	Low complication rates in both groups	High satisfaction, slightly higher in transaxillary group	Endoscopic transaxillary approach offers reliable aesthetic outcomes with high satisfaction, especially for patients seeking minimal visible scarring.
Aygit AC, Basaran K, Mercan ES. [35]	Transaxillary Subfascial	Inconspicuous scar hidden in axilla	Minimal due to avoidance of muscle dissection	Low (16% developed Baker grade II capsular contracture)	Exceptionally high (96% very satisfied)	The technique effectively eliminates visible breast scars, minimizes pain, and achieves high patient satisfaction with a low complication rate.
Esposito E et al. [36]	Endoscopic Video-Assisted Single-Port	Optimized by placing incision in axillary fold	Well-managed with preoperative blocks	No major complications reported	High satisfaction due to absence of adverse events	Technique is highly effective in minimizing visible scarring and managing postoperative pain, with no significant complications reported.
Maximiliano J et al. [37]	Various Techniques for Augmentation/Mastopexy	Grades 4 or 5 in 61% of isolated augmentation cases	N/A	14% reintervention rate, no significant differences based on dissection plane	High satisfaction, no significant differences in outcomes	Emphasizes the importance of precise surgical technique, with consistent results across different approaches.
Stümpfle RL et al. [38]	Transaxillary Revision with Muscle-Splitting	Excellent, no major complications observed	N/A	No major complications, 100% satisfaction	High satisfaction with scar quality and overall results	Transaxillary approach for revision surgery is safe and effective, offering good upper and medial pole coverage with adequate lower pole expansion.
Niechajev Igor [39]	Transaxillary Breast Augmentation (TBA)	Well-concealed, aesthetically pleasing scars, with 7% experiencing unsightly scars	Higher pain levels, managed with anesthetics	Complications such as unsightly scars (7%) were resolved with technique modifications	High satisfaction, particularly with scar quality and symmetry	TBA offers superior aesthetic outcomes for patients desiring discreet augmentation, though it requires a longer learning curve and close monitoring.

A study by Ana Claudia Weck Roxo [32] compared the outcomes of transaxillary augmentation mammaplasty performed with and without endoscopic assistance in 20 female patients. The mean operative time was longer in the endoscopic group (2 hours 5 minutes) compared to the non-endoscopic group (1 hour 15 minutes). In terms of scar quality, both groups had high levels of satisfaction, with the endoscopic group reporting 30% very satisfied and 50% satisfied patients, while the non-endoscopic group had 60% very satisfied and 30% satisfied patients. Postoperative pain was slightly higher in the non-endoscopic group, with 70% experiencing grade 3 pain or higher, compared to 60% in the endoscopic group. Regarding complications, both groups had minimal issues, with one minor complication (hematoma and wound dehiscence) reported in each group. Postoperative satisfaction was comparable between the groups, with 70% of the non-endoscopic and 70% of the endoscopic group reporting being very satisfied or satisfied with the aesthetic results. There were no significant differences in the overall outcomes, suggesting that while endoscopic assistance prolongs operative time, it does not significantly improve postoperative results or reduce complications [32].

The study by Philippe A. Giordano et al. [33] on endoscopic transaxillary breast augmentation, conducted between November 1996 and December 2005, assessed 306 patients with an average age of 38 years. The transaxillary approach was used to achieve hidden scars, with no reported concerns about scarring quality, which effectively remained concealed within the axilla. The average implant size was 290 cc, with about 60% placed submusculofascially. Postoperative pain was minimized due to the avoidance of muscle dissection, leading to a smoother recovery period, with most patients experiencing minimal discomfort. The overall patient satisfaction was high, supported by a low complication rate of 6.5%. Complications were relatively low, with a 6.2% reoperation rate. Malposition occurred in 9 patients (3%), capsular contracture (Baker III or IV) in 5 patients (1.6%), and hematomas requiring evacuation in 3 patients (1%). Other complications included one case each of postoperative infection (0.3%), pneumothorax (0.3%), and hypertrophic scar (0.3%). Additionally, patient follow-up revealed consistent satisfaction, with the technique effectively addressing minor to moderate breast deformities while preserving aesthetic outcomes.

Similarly, in a study by Sim HB in 2014. [31] focused on transaxillary breast augmentation using sharp electrocautery dissection under direct endoscopic vision. The findings highlighted that this method offers significant benefits, particularly in terms of scar quality, postoperative pain, and patient satisfaction. The transaxillary approach effectively avoided visible scars on the breast, which is particularly important for East Asian women who are prone to noticeable scarring. Over 60% of breast augmentations in the study were performed via the axilla, with no reported issues related to scar quality. The sharp electrocautery dissection minimized tissue damage, leading to reduced postoperative pain. About 84% of patients (174 out of 232) returned to normal life within three days post-surgery, and all patients were back to normal within seven days, indicating a quick recovery. The technique resulted in high levels of patient satisfaction, with no major complications such as severe bleeding, infection, or implant-related issues reported. Capsular contracture occurred in only 2.16% of patients (5 out of 232), with no instances in patients with anatomical implants. This low complication rate and effective aesthetic outcomes contributed to overall positive patient satisfaction [31].

Momeni A et al. [34] compared the outcomes of 76 patients who underwent primary augmentation mammaplasty using two different approaches: the inframammary approach (38.2%) and the endoscopic transaxillary approach (61.8%). The endoscopic transaxillary approach offered a significant advantage in scar quality by placing the incision in the armpit, leaving the breast area scar-free, which is particularly desirable for patients seeking minimal visible scarring. Both approaches resulted in minimal postoperative complications, with a low overall complication rate of 10.6% for the transaxillary approach and 10.3% for the inframammary approach. There were no instances of hematoma, infection, or rippling reported in the transaxillary group, indicating effective postoperative management and patient recovery. Patient satisfaction, measured using the CSQ-8 questionnaire, was high for both groups, with median scores of 31 for the transaxillary group and 30 for the inframammary group. Although the difference was not statistically significant, the transaxillary approach showed a slight trend toward higher satisfaction. Overall, the study concluded that the endoscopic transaxillary approach is a safe and effective method, offering reliable aesthetic outcomes and high patient satisfaction [34].

The study by Aygit AC, Basaran K, and Mercan ES. [35] on transaxillary total subfascial breast augmentation highlighted several key outcomes: The transaxillary approach resulted in an inconspicuous scar hidden in the axilla, with none of the patients reporting concerns about scarring. This method effectively eliminates visible breast scars, offering a significant cosmetic advantage. The procedure avoided muscle dissection, a common cause of postoperative pain in other techniques. As a result, patients experienced less discomfort, and all but one were discharged the day after surgery. The minimal pain reported aligns with the conservative approach to nerve and tissue handling during the procedure. Patient satisfaction was exceptionally high, with 96% of the patients expressing they were "very satisfied" with their outcomes using a 0-10 point scoring system. Only 16% of patients developed Baker grade II capsular contracture, a low complication rate that supports the effectiveness of the subfascial technique. Additionally, there were no complaints of nipple hypoesthesia or implant malposition, further contributing to positive patient experiences [35].

Esposito E et al. [36], who performed a retrospective analysis of 16 patients at the National Cancer Institute of Naples who underwent endoscopic video-assisted single-port breast reconstruction following mastectomy, revealed promising outcomes. The mean patient age was 56 years, with an average implant volume of 440 cc. The procedure had a fast learning curve, with a mean operative time of 134 minutes. Scar quality was optimized by placing the incision within the axillary fold, effectively hiding it. Postoperative pain was well-managed with preoperative blocks (ESP and PECS), contributing to the absence of major complications. The study reported no instances of seroma, hematoma, infection, or skin dehiscence, and all patients were complication-free during the median follow-up period of eight months. Patient satisfaction was high, reflected in the successful outcomes and absence of adverse events. Overall, this technique demonstrated a high level of efficacy in minimizing visible scarring and managing postoperative pain, with no significant complications reported, making it a viable option for breast reconstruction in selected patients [36].

Similarly, a retrospective cohort study by Maximiliano J et al. [37] analyzed the outcomes of 50 women (100 breasts) who underwent breast augmentation or mastopexy associated with breast implants between 2011 and 2016. The study focused on scar quality, postoperative pain, patient satisfaction, and complications. Scar quality was assessed using the Stony Brook Scar Evaluation Scale, with 61% of isolated breast augmentation cases achieving grades 4 or 5, compared to 53% in the mastopexy with implant group. Postoperative complications included a 14% reintervention rate, with causes such as seroma (4%), asymmetry (4%), capsular contracture (3%), and hematoma (1%). The study found no statistically significant differences in complications or reintervention rates based on surgical planning, implant shape, or dissection plane. However, the subglandular plane had a 14% reintervention rate, while the double plane had a 19% rate, and no reinterventions occurred with subfascial planes. Despite the lack of significant differences in outcomes, the study emphasized the importance of precise surgical technique and routine in achieving optimal results, consistent with findings from larger centers and other studies [37].

Stümpfle RL et al. [38] reported a retrospective study of 41 women who underwent transaxillary revision breast augmentation with conversion to a muscle-splitting plane. The results demonstrated excellent outcomes in terms of scar quality, postoperative satisfaction, and complication rates. The average patient age was 38 years, with a mean BMI of 21.9 kg/m². The primary indications for revision were implant rippling and the desire for larger implants, with new implant sizes ranging from 325cc to 430cc. The mean operative time was 53 minutes, with a follow-up period averaging 13 months. Patient satisfaction was high, with 100% of participants reporting they were pleased with both the overall results and the scar quality. No major complications were observed. These findings suggest that the transaxillary approach for muscle-splitting breast augmentation revision surgery is a safe and effective technique, offering good upper and medial pole coverage with adequate lower pole expansion without increasing operative time or costs. The study supports the use of this technique for patients seeking revision surgery after initial transaxillary breast augmentation [38].

A study by Niechajev Igor [39] analyzed the outcomes of transaxillary breast augmentation (TBA) in 140 patients between 1988 and 2009, with a focus on scar quality, postoperative pain, and patient satisfaction. The study revealed that the axillary incision, particularly when modified to the "boomerang" design, resulted in well-concealed scars that patients found aesthetically pleasing. In most cases, the scars were practically invisible, even when patients raised their arms. However, 7% of patients experienced unsightly scars, often due to complications such as axillary folliculitis or suture intolerance. These issues were resolved in later years through modifications in incision design and suture materials. Overall, the high satisfaction with the axillary scar was a significant advantage of TBA, particularly for patients concerned about visible scars on the breast. In terms of postoperative pain, TBA was associated with higher pain levels compared to other techniques, such as the submammary approach. However, the implementation of a topical, long-acting anesthetic regimen significantly reduced the pain, allowing for the use of fewer potent analgesics, including opioids. Continuous regional analgesia, provided through an epidural catheter, further enhanced pain management. Despite the initial discomfort, patients generally tolerated the procedure well, with pain management strategies making TBA a viable option for those desiring scar-free breast augmentation. Patient satisfaction with TBA was high, particularly regarding the absence of visible scars on the breast and the natural aesthetic results achieved with the procedure. The study reported that all patients with anatomic implants experienced good symmetry, with no instances of implant rotation. The use of the "double-lubrication" technique and specialized instruments, such as the breast implant pusher, improved the surgical process and contributed to patient satisfaction. While TBA requires a longer learning curve and closer postoperative monitoring, the study concluded that with proper patient selection and technique, the aesthetic outcomes were superior to those of other breast augmentation methods, particularly for patients with minor breast asymmetries and those wishing to keep their augmentation discreet [39-40].

Periareolar technique: Periareolar breast augmentation is a surgical technique used to enhance breast size and shape through an incision made along the border of the areola, the pigmented area surrounding the nipple. This method offers several advantages, including precise control over the creation of the implant pocket and minimal visible scarring [41]. The pectoralis major muscle fibers can be carefully released when necessary, allowing the surgeon to work in both the subglandular and subfascial planes with ease [42,43]. The incision placement allows the surgeon to perform the procedure under direct vision, ensuring accurate development of the subglandular, subfascial, or submuscular planes. Additionally, this technique provides a controlled release of pectoralis major muscle fibers when needed, allowing for a customized approach to achieving the desired breast contour [44,45]. The periareolar incision is often preferred due to its ability to create a subtle, inconspicuous scar that typically heals to an imperceptible fine line. This method is ideal for patients who prioritize minimal scarring and are comfortable with a scar that is only visible when the breast is fully exposed [41]. Table 3 describes the various studies reporting patient satisfaction and scar quality on the periareolar technique.

**Table 3:** Summary of studies on peri-areolar techniques of breast augmentation VAS: Visual analog scale [40, 45-47]

Study	Scar Quality	Postoperative Pain and Complications	Patient Satisfaction
Klinger M et al. [45]	Areolar widening (2-5%), hypertrophic or pinched scars (1-5.8%)	Low incidence of complications; postoperative pain modest; need for outpatient secondary surgery low (9%)	High satisfaction with mean VAS scores 7.9-8.4; aesthetic scores 4.0-4.6 out of 5
Moltó-García R et al. [46]	Generally acceptable; one instance of periareolar scar widening	Minimal complications; one case (1.7%) of wound infection, one case (1.7%) of partial periareolar necrosis	71.43% very positive; 14.29% moderately satisfying; 10.71% neutral; 3.57% dissatisfied
Muhammad Humayun Mohmand et al. [40]	Minimal complications; 2 patients (6.3%) with slightly stretched scars	Temporary sensation change in 21.9% at 3 months, reduced to 6.3% at 6 months; no implant infections or capsular contracture	High satisfaction; no implant infections, removals, or capsular contracture; no visible deformities
Byun IH [47]	Generally favorable; 9.02 satisfaction score on Likert scale; 3 patients (1.55%) with hypertrophic scars	Minimal complications; 1 case (0.52%) of hematoma, 2 cases (1.04%) of capsular contracture	High satisfaction due to specific nipple positioning technique and minimal scarring

The study by Klinger M et al. [46] evaluated 4,502 female patients undergoing 5,028 consecutive breast procedures using the periareolar approach. The procedures included mastopexy, breast augmentation with mastopexy, secondary breast augmentation, correction of stenotic and tuberous breasts, lumpectomies, and breast reductions. In terms of scar quality, the results showed a relatively low incidence of poor scar outcomes, with areolar widening occurring in 2-5% of cases and hypertrophic or pinched scars in 1-5.8% of cases, depending on the procedure. The technique generally resulted in satisfactory scarring due to the use of precise suturing techniques and careful postoperative management. Regarding patient satisfaction, the study reported high satisfaction scores, with mean visual analog scale (VAS) scores ranging from 7.9 to 8.4, depending on the procedure. Surgeons' aesthetic outcome scores were similarly high, with mean ratings between 4.0 and 4.6 out of 5. Additionally, the study noted that postoperative pain and complications were modest and decreased over time as the surgical team's experience grew. The need for outpatient secondary surgery, such as scar revision, was relatively low (9% of the 1,400 patients analyzed). The authors highlighted the versatility of the periareolar approach for various breast conditions, although it was not suitable for severe hypertrophy or ptosis cases with excessive or inelastic skin, where longer scars were preferred [46].

Similarly, a study by Moltó-García R et al. [47] assessed 56 cases of periareolar augmentation mastopexies performed over three years, focusing on patients aged 25 to 45. The results highlighted high levels of patient satisfaction, with 71.43% of patients rating their outcomes as very positive and 14.29% describing them as moderately satisfying. Only 10.71% were neutral about their results, while 3.57% were dissatisfied due to complications such as the "waterfall" effect after weight loss and the widening of periareolar scars, both requiring surgical correction. Scar quality was generally acceptable, though there was one instance of periareolar scar widening. Postoperative complications were minimal, with no major infections reported and a single case (1.7%) of wound infection that was resolved with antibiotics and minor surgical intervention. There was also one case of partial periareolar necrosis (1.7%) in a heavy smoker, which required debridement and delayed closure but resulted in no significant aesthetic issues. The study emphasized that many women presenting for breast augmentation or mastopexy might have undiagnosed tuberous breasts. The new approach developed during the study has improved surgical outcomes by adapting the technique based on these observations, leading to better overall patient satisfaction and results [47].

Interestingly, another study by Muhammad Humayun Mohmand and Muhammad Ahmad [41], conducted from 2004 to 2010, involved 32 patients undergoing periareolar breast augmentation. The average patient age was 30.7 years, with the majority (71.9%) being 30 years or younger. The mean incision length was 5.8 cm, and implant sizes varied, with most patients receiving implants larger than 305 ml. Postoperative outcomes were generally positive, with high patient satisfaction and minimal complications. Only two patients (6.3%) experienced slightly stretched scars, and no cases of implant infection, removal, or capsular contracture were reported. A temporary change in sensation was noted in 21.9% of patients at three months post-operation, reducing to 6.3% at six months. No cases of rippling or other visible deformities were observed. The study highlighted the advantages of the extra-glandular periareolar approach, which minimizes the risk of capsular contracture by avoiding breast tissue contamination during surgery. This technique also allows for the correction of nipple-areola complex asymmetry. The study emphasized the importance of surgical expertise in achieving optimal outcomes with the periareolar approach, which provides a favorable balance between effective augmentation and reduced risk of complications [41].

The study by Byun IH [48] reviewed 193 patients who underwent periareolar augmentation mastopexy between January 2019 and December 2021, focusing on scar quality, postoperative pain, and patient satisfaction. The average age was 43.19 years, with a mean BMI of 23.33. Scar quality was generally favorable, with a 9.02 satisfaction score on the Likert scale. However, three patients (1.55%) developed hypertrophic scars, which required triamcinolone injections or scar revision. Postoperative complications were minimal, with only one case (0.52%) of hematoma and two cases (1.04%) of capsular contracture being successfully treated. The study emphasized the importance of careful surgical planning and patient selection to achieve stable, satisfactory outcomes with low complication rates. The specific nipple positioning technique, which placed the areola 3-4 cm above the inframammary fold (IMF), contributed to patient satisfaction by creating aesthetically pleasing results. Despite the potential challenges, the periareolar approach provided a balance between effective mastopexy and minimal visible scarring, making it a preferred choice for many patients [48].

Transumbilical breast augmentation: Transumbilical breast augmentation (TUBA), developed in 1993 by Johnson and Christ, is a surgical technique used for breast augmentation that involves inserting breast implants through an incision made in the navel (umbilicus) [49,50]. This method offers several advantages over other incision sites, including the absence of visible scarring on the breast, reduced tension on the incision, and potentially lower levels of postoperative pain. TUBA allows for precise shaping of the implant pocket, which can help achieve better symmetry and prevent complications such as symmastia. Additionally, the technique minimizes the risk of infection and hematoma by avoiding dead space during pocket formation. However, TUBA also has its disadvantages. It currently does not support the use of prefilled implants, whether saline or silicone and requires specialized equipment and extensive training for optimal results. Surgeons must undergo formal training to perform TUBA safely and effectively, as the procedure demands specific skills and knowledge not covered in general surgical education. Despite these challenges, TUBA has been proven to be a safe and effective option for breast augmentation when performed by well-trained professionals [51]. Table 4 depicts the various studies included in this article describing TUBA.

**Table 4:** Summary of studies on TUBA approach of breast augmentation TUBA: Transumbilical breast augmentation; CSQ: Clinical satisfaction questionnaire [51-54]

Study	Participants	Procedures	Outcomes	Complications	Patient Satisfaction
Huang et al. [51]	74 patients, mean age 36.4 years	TUBA with prefilled silicone implants	Feasible for all dissection planes and implant types; satisfactory aesthetic results	4.6% major revision surgeries in endoscope-assisted group; none in full-endoscopy group	High satisfaction, especially in full-endoscopy group; no major scarring concerns
Brennan WA et al. [52]	245 patients	TUBA	Minimal scar complications; low postoperative pain; high patient satisfaction	Local wound infection (3.2%); no implant infections reported	79.5% very satisfied; high satisfaction correlated with retropectoral placement
Won Lee et al. [53]	42 women, aged 23 to 46 years (average 31.6 years)	TUBA with cohesive silicone gel implants	Successful outcomes with minimal scarring; high satisfaction	Hypertrophic scarring in 1 patient (2.3%); transient periumbilical bulging in 5 patients (11.9%); capsular contracture in 3 out of 84 breasts (3.5%)	High satisfaction; minimal postoperative pain
Won Lee et al. [54]	69 women and 1 man, implants ranging 225 to 300 mL	TUBA with cohesive silicone gel implants	Effective with minimal scarring and quick recovery; no visible breast scars	One patient experienced abdominal bulging; no other significant complications reported	Very high satisfaction; minimal postoperative pain; all but one patient content with results
Riggio et al. [55]	4 patients undergoing transabdominal breast augmentation	Combined with abdominoplasty	Favourable scar quality; high satisfaction; improvements in body image	Not extensively detailed, but minimal complications implied due to the less invasive nature	High satisfaction, as reflected in the Clinical Satisfaction Questionnaire (CSQ-8); positive feedback on minimal visible scarring and cosmetic improvements

The study by Huang et al. [52], evaluated the feasibility and outcomes of transumbilical silicone implant breast augmentation (TUSBA) using various types of prefilled silicone implants. Conducted between June 2016 and April 2021, the study retrospectively reviewed 74 patients, with a mean age of 36.4 years, who underwent TUSBA. The implants used had smooth, textured, or nanotextured surfaces and ranged up to 500 mL in size. Two groups were analyzed: an endoscope-assisted group and a full-endoscopy group. The endoscope-assisted group initially used conventional TUBA techniques modified for prefilled implants, while the full-endoscopy group utilized endoscopic monitoring for the entire procedure. The study found that TUSBA is feasible for all dissection planes (subglandular, subfascial, dual-plane) and adaptable to various implant types. Results indicated no excessive postoperative pain in either the breast or abdomen for all patients, reflecting a positive outcome in terms of postoperative discomfort. Aesthetic results were satisfactory across both groups, with no major scarring concerns. However, the endoscope-assisted group had a higher complication rate, with 4.6% of patients requiring major revision surgeries, compared to none in the full-endoscopy group. Overall, the study suggests that TUSBA with full endoscopic monitoring is a viable and effective method for silicone implant breast augmentation, offering reduced complications and high patient satisfaction [52].

The study on transumbilical breast augmentation (TUBA) by Brennan WA and Haiavy J. [52] evaluated 245 patients and provided insights into scar quality, postoperative pain, and patient satisfaction. Scar quality was generally positive, with minimal complications related to the umbilical incision. A small percentage experienced local wound infection (3.2%) or scar complications, but no implant infections were reported. Postoperative pain was relatively low, with patients typically requiring narcotics for only 48-72 hours. The average operating room time was 43.9 minutes, and the procedure predominantly used retropectoral placement (84.9%). Patient satisfaction was high, with 79.5% of patients very satisfied with their results. The study found no significant correlation between patient satisfaction and factors like implant size, operating room time, or sensory disturbances. However, satisfaction was positively correlated with retropectoral implant placement (p = 0.0097) and negatively with prepectoral placement (p = 0.0097) and perceived firmness (p < 0.0001). Overall, the study concluded that TUBA is a safe technique with high patient satisfaction and minimal complications [52].

Similarly, another study by Won Lee et al. [53] assessed the outcomes of transumbilical breast augmentation using cohesive silicone gel implants in a subpectoral plane, an innovative approach that expands the implant options for this technique. Conducted between June 2011 and January 2012, the study included 42 women aged 23 to 46 years (average age 31.6 years) with mammary hypoplasia without ptosis, seeking augmentation with minimal scarring and a natural feel. Results demonstrated that the transumbilical approach was highly successful in achieving desired outcomes with prefilled silicone implants. Most patients reported satisfactory results with minimal postoperative pain. Scar quality was generally favorable, with only one patient (2.3%) experiencing hypertrophic scarring requiring revision. Complications were minimal, including transient periumbilical bulging in 5 patients (11.9%) and conversion to another incision due to capsular contracture in 3 out of 84 breasts (3.5%). Overall, the study suggests that this transumbilical technique is a safe and effective option for breast augmentation, providing a scarless alternative with good patient satisfaction [53].

Interestingly, a study by Won Lee and Seong Hwan Kim [54] evaluated the outcomes of transumbilical silicone breast augmentation (TUSBA) over a 13-year period, with a focused analysis of patient outcomes over the past five years. Conducted between January 2018 and December 2022, this study included 69 women and one man who underwent breast augmentation with cohesive silicone gel implants placed in the subpectoral pocket. Implant sizes ranged from 225 to 300 mL, with an average of 272 mL. Two patients received implants of different sizes to address breast asymmetry. The results demonstrated that TUSBA effectively provides minimal scarring, as the incision is made through the umbilicus, resulting in no visible breast scars. Patients reported minimal postoperative pain, contributing to a quicker recovery and the ability to maintain unrestricted arm movement shortly after surgery. Overall, patient satisfaction was very high, with all but one patient expressing contentment with the aesthetic results. The single dissatisfied patient experienced abdominal bulging postoperatively. The study suggests that TUSBA is a viable option for those seeking breast augmentation, offering aesthetic and functional benefits with a low complication rate [54].

Additionally, a study by Riggio et al. [55] evaluated four cases involving transabdominal (transumbilical-like) breast augmentation in conjunction with abdominoplasty. Scar quality was notably favorable, as the use of the abdominoplasty incision allowed for a scarless approach to the breast tissue, preserving aesthetic outcomes. Postoperative pain was not extensively detailed, but the focus on minimizing visible scarring suggests a generally favorable pain profile consistent with less invasive procedures. Patient satisfaction was high, as reflected in the Clinical Satisfaction Questionnaire (CSQ-8) administered during long-term follow-up (37 months). The study found that patients, three undergoing postmastectomy reconstruction with transverse rectus abdominis myocutaneous (TRAM) flaps and one undergoing cosmetic abdominoplasty, reported improvements in body image and satisfaction with their contralateral breast augmentation. The combined approach of using a transabdominal incision and augmenting the breast without additional scarring on the breast itself received positive feedback, highlighting the benefits of minimizing visible scarring and achieving cosmetic and psychosocial improvements [55].

Complications based on types of implants

Breast augmentation involves various methods such as implants, injectable materials, and autologous fat grafting. While these techniques have significantly advanced over time, they are not without complications. The use of injectable materials such as paraffin, silicone, and hydrophilic polyacrylamide hydrogel has historically resulted in numerous complications, ranging from skin necrosis and infection to severe cutaneous reactions years after injection. Modern breast implants, particularly silicone-based ones, have seen improvements in safety and durability. However, complications such as implant rupture, capsular contracture, and silicone migration continue to pose risks, often necessitating further surgical intervention. Sensory alterations, striae distensae (stretch marks), and rare conditions like Mondor’s disease (a benign thrombophlebitis) have also been reported following augmentation procedures. Additionally, breast implant-associated anaplastic large cell lymphoma (biALCL), a rare type of T-cell lymphoma, has been linked to textured implants. Lastly, autologous fat grafting, though generally safer with a lower complication rate, can still lead to cutaneous complications such as erythema, cysts, and abscess formation. Understanding these risks is crucial for both patients and surgeons in planning and managing breast augmentation procedures [56].

In a retrospective study by Hidalgo DA. [57], of 220 breast augmentation patients, complications related to the incision site, implant variables, and pocket plane selection were reviewed. Among 77 primary augmentations and 80 unilateral augmentations for symmetry in breast reconstruction, there were 11 revisions due to unilateral malposition, changes in implant shape, or size. Additionally, four cases involved saline implant deflation requiring replacement, and four conversions from saline to silicone gel implants were performed. In 63 secondary augmentation cases, two patients developed hematomas, and two experienced infections, both requiring implant removal and subsequent replacement. The study focused on surgical techniques and recommendations associated with these three variables without addressing long-term issues like capsular contracture or saline implant deflation rates. Overall, the complications highlight the importance of careful surgical planning and selection of appropriate techniques to minimize risks in breast augmentation procedures [57].

Similarly, in a prospective outcome study by Swanson E. [58], on 225 breast augmentation patients, the reported complication rate was 10.2%. Common issues included nipple numbness, which affected 39.1% of patients, though persistent numbness was rare, occurring in only 2.3%. Other complications were not detailed in the summary but contributed to the overall complication rate. The study found a significant correlation between lower complication rates and higher patient satisfaction with the results. Despite the complications, the vast majority of patients were satisfied with the surgery, with 98.7% indicating they would choose to undergo the procedure again. Overall, the study highlights the importance of managing complications to maintain high patient satisfaction in breast augmentation [58].

A systematic review by Wong CH et al. [59] aimed to assess the impact of textured implants versus smooth implants on capsular contracture rates in subglandular breast augmentation. They analyzed data from six randomized controlled trials involving 235 patients (470 breasts). The review found that textured implants were associated with a significantly lower incidence of capsular contracture, as indicated by Baker grade evaluations at 1 year (relative risk, 4.16), 3 years (relative risk, 7.25), and 7 years (relative risk, 2.98) of follow-up. Despite these findings, applanation tonometry, used as an objective measure of firmness, did not show significant differences between textured and smooth implants (weighted mean difference, -1.54). The self-assessment questionnaires revealed that while capsular contracture is a critical factor, it is just one aspect of overall patient satisfaction. Overall, the study suggests that textured implants reduce the incidence of early capsular contracture in subglandular breast augmentation. However, the authors call for additional research to further investigate the long-term effects and confirm the sustained benefits of implant texturization [59].

Similarly, another systematic review by Hedström K et al. [60] included 16 studies on breast augmentation using dermal fillers containing copolyamide involving 196 women. A total of 333 complications were reported across these studies. The majority of complications were long-term, occurring between 1 and 60 months post-injection, with a median onset of 18 months. The most common long-term complications included nodules in the breast (130 cases), pain (92 cases), inflammation or infection (43 cases), breast deformities such as volume loss or swelling (35 cases), and distant migration of the filler (23 cases). Short-term complications, which occurred within 30 days of injection, were rarely reported. In 12 of the 16 studies, dermal filler migration was noted, both locally within the breast and to distant sites such as the abdominal wall, pubic area, and thoracic wall. Treatment of complications often involved surgical intervention, with 92% of affected women requiring surgical removal of the filler or breast incision. The review highlighted significant safety concerns due to the high frequency of severe complications, recommending against the use of copolyamide-based dermal fillers for breast augmentation until further long-term safety data are available [60].

Interestingly, a study by Liu C et al. [61] compared complication rates following breast augmentation between transfeminine patients and cisgender women. A total of 1864 transfeminine patients from 14 studies were analyzed, focusing on primary outcomes such as complications, patient satisfaction, and reoperation rates. Among transfeminine patients, the pooled complication rates were as follows: capsular contracture at 3.62%, hematoma or seroma at 0.63%, infection at 0.08%, and implant asymmetry or malposition at 3.89%. When comparing these rates with those historically reported for cisgender women, the study found no significant difference in the rates of capsular contracture (p = 0.41) and infection (p = 0.71) between the two groups. However, transfeminine patients had significantly higher rates of hematoma or seroma (p = 0.0095) and implant asymmetry or malposition (p < 0.00001). These findings suggest that while breast augmentation is generally safe and effective for transfeminine individuals, there is an increased risk of certain complications, such as hematoma, seroma, and implant malposition, compared to cisgender women. This highlights the need for careful surgical planning and patient counseling in transfeminine breast augmentation procedures [61].

Another study by Calobrace MB et al. [62] analyzed long-term data from 2,565 primary breast augmentation patients to identify risk factors associated with capsular contracture, the most common complication following breast implant surgery. Among 5,122 implants studied, 333 capsular contracture events were reported in 224 patients, with an overall Kaplan-Meier rate of 10.8% over ten years. The study used multivariate analysis to identify eight significant risk factors for capsular contracture, such as implant placement, implant surface, incision site, development of hematoma or seroma, device size, use of a surgical bra, and the types of pocket irrigation with steroids or antibiotics (all p-values < 0.05). Additionally, the study found that certain factors were more strongly associated with early-onset capsular contracture, particularly those occurring within two to five years post-implantation. Notably, implants with a smooth surface and the use of steroid pocket irrigation were linked to a higher risk of early capsular contracture. Surgical factors, such as periareolar incision and subglandular placement, also contributed to increased risk, while smaller device sizes and antibiotic irrigation were significant device-related factors. The development of hematoma or seroma and the use of a surgical bra were also associated with a higher incidence of capsular contracture. These findings underscore the importance of considering both patient-specific and surgical factors in reducing the risk of capsular contracture after breast augmentation [62].

Similarly, another systematic review and meta-analysis assessed the safety and complication rates of single-stage augmentation-mastopexy, a procedure that simultaneously increases breast volume and reduces the skin envelope. A total of 23 studies met the inclusion criteria, encompassing 4,856 cases. The average follow-up period ranged from 16 to 173 weeks, with most studies having a follow-up of less than one year. The overall pooled complication rate for single-stage augmentation-mastopexy was 13.1%, with the most common complication being recurrent ptosis at 5.2%. Poor scarring was the second most common complication, with an incidence rate of 3.7%. Capsular contracture and tissue-related asymmetry had similar rates of 3.0% and 2.9%, respectively. The incidences of infection, hematoma, and seroma were each below 2%, indicating they are relatively rare complications. Additional findings from the review include a reoperation rate of 10.7%, as reported in 13 studies. Patient satisfaction data, available from three studies, suggests overall positive outcomes. The review highlights the significant heterogeneity across studies due to differences in surgical techniques, outcome definitions, and follow-up durations. Despite these variations, the analysis suggests that, with careful patient selection, the complication and reoperation rates for single-stage augmentation-mastopexy are acceptably low [63].

A systematic review and meta-analysis examined complications associated with acellular dermal matrix (ADM)-assisted breast reconstruction compared to non-ADM procedures. From an initial 61 abstracts, 16 studies met the inclusion criteria and were analyzed for pooled complication rates. The most common complication observed was skin flap necrosis, with an estimated pooled rate of 10.9%, significantly higher than rates in non-ADM procedures. Seroma followed, with a rate of 6.9%, while infection and cellulitis rates were 5.7% and 2.0%, respectively. Hematoma had a lower incidence of 1.3%. ADM-assisted reconstructions also showed a lower rate of capsular contracture (0.58%) compared to traditional tissue expander/implant (TE/I) reconstructions, which have rates ranging from 3% to 18%. The meta-analysis indicated that ADM use is associated with a higher risk of certain complications, such as seroma, infection, and reconstructive failure, compared to non-ADM procedures. Notably, ADM-assisted reconstructions were nearly four times more likely to develop seroma and three times more likely to experience infection and reconstructive failure. Although ADM seems to reduce the risk of capsular contracture, the increased likelihood of other complications suggests a need for cautious application of ADM in breast reconstruction, highlighting the importance of tailored surgical strategies and further research to optimize outcomes [64].

Fascinatingly, a study by Pires GR et al. [65] aimed to determine the complication rates and failure of prepectoral breast reconstruction using different types of acellular dermal matrices (ADMs), including human (hADM), porcine (pADM), and bovine (bADM) ADMs. A total of 33 studies met the inclusion criteria, examining 6,046 prepectoral reconstructions from January 2010 to August 2021. The overall implant loss rate was similar across the different types of ADMs, with pADM and hADM both at 4.0% and bADM at 3.7%. However, the study found that bovine ADM had the highest rates of several complications compared to hADM and pADM. Specifically, bADM showed the highest rates of capsular contracture (6.1%), infection (9.0%), skin flap necrosis (8.3%), dehiscence (5.4%), and hematoma (6.1%). Human ADM had the highest rate of postoperative seroma at 5.3%, followed by pADM at 4.6% and bADM at 4.5%. Overall, the findings suggest that while prepectoral breast reconstruction using ADM generally results in relatively low complication rates, bovine ADM is associated with a higher proportion of complications in several categories. These results indicate that the choice of ADM may influence the complication profile, particularly in prepectoral breast reconstruction [65].

Another study by Nolan IT et al. [66] aimed to compare the complication rates in prepectoral breast reconstruction with and without the use of acellular dermal matrices (ADMs). The systematic review and meta-analysis included 515 reconstructions from four studies, primarily involving nipple-sparing mastectomies and tissue-expander reconstructions. The analysis found no significant difference in the overall rate of complications between reconstructions performed with ADM and those without ADM. In terms of short-term complications, the rates of reconstructive failure were slightly lower in the ADM cohort (1.2%) compared to the non-ADM cohort (2.8%). The incidence of seroma was significantly lower in the ADM group (1.2%) versus the no-ADM group (8.3%). Other short-term complications, such as hematoma (1.2% vs. 2.1%), infection (4.7% vs. 4.2%), and mastectomy flap ischemia or necrosis (2.4% vs. 5.2%), were comparable between the two groups. Long-term complications included rippling, which was observed in 3.3% of the ADM group and 5.1% of the no-ADM group, and capsular contracture, which was higher in the ADM group (6.8%) compared to the no-ADM group (3.4%). Overall, the study suggests that there is no significant difference in complication rates between prepectoral breast reconstructions with and without ADM. The decision to use ADM should be based on individual clinical scenarios and surgeon discretion [66].

Additionally, a meta-analysis aimed to update evidence on the use of acellular dermal matrix (ADM) in tissue expander/implant-based (TE/I-based) breast reconstruction by assessing its impact on postoperative complications. The analysis included 11 studies published between January 2010 and February 2015, comparing the incidence of complications in TE/I-based breast reconstructions with and without ADM. The complications were categorized into overall complications, infection, hematoma/seroma, and explantation. The study findings indicated that the use of ADM in TE/I-based breast reconstruction was associated with a higher risk of overall complications, with an odds ratio (OR) of 1.33 (95% CI 1.03-1.70, p = 0.03). The rates of infection (OR = 1.47, 95% CI 1.04-2.06, p = 0.03) and hematoma/seroma (OR = 1.66, 95% CI 1.13-2.44, p = 0.01) were also significantly increased in the ADM group compared to the control group. However, there was no significant difference in the rate of explantation between the two groups (OR = 1.37, 95% CI 0.89-2.11, p = 0.15). The meta-analysis concludes that the use of ADM in TE/I-based breast reconstruction increases the incidence of certain complications, specifically overall complications, infection, and hematoma/seroma. Despite these findings, the evidence quality was rated as level C, indicating the need for caution in clinical application. The study suggests more high-quality, large-sample studies to further clarify the impact of ADM on postoperative outcomes [67].

Another prospective, multicentre cohort study by Venturi ML et al. [68] evaluated the use of sterile human acellular dermal matrix (ADM) in immediate expander-based breast reconstruction, focusing on complication rates, particularly infection and seroma, and the impact of the sterilization process on graft incorporation. Over a 1-year period, 65 consecutive breast reconstructions were performed using a sterile human matrix, following a standardized protocol. The study aimed to determine whether sterile ADM provided a more favorable risk profile compared to non-sterilized ADM in terms of postoperative complications. The results showed a low complication rate of 4.6 percent across all cases. Among the complications, there was one instance of cellulitis (1.5 percent) and two cases of partial mastectomy flap necrosis (3.0 percent) that required debridement. Importantly, there were no reports of seroma or explanation in any of the cases. Additionally, graft incorporation was successful in all reconstructions, as confirmed histologically in the first 20 biopsies. The study concluded that the use of sterile human ADM in immediate expander-based breast reconstruction results in a low rate of complications and reliable graft incorporation. Moreover, the sterilization process did not adversely affect the integration of the graft. These findings suggest that sterile ADM may offer a more favorable complication profile compared to nonsterilized ADM, particularly in terms of reducing infection and seroma rates [68].

Discussion

The studies on inframammary fold (IMF), subpectoral, and prepectoral breast augmentation techniques reveal significant differences in postoperative pain and patient satisfaction. Subpectoral augmentation, while effective, often results in higher postoperative pain due to muscle manipulation, leading to discomfort and potential animation deformity. In contrast, prepectoral augmentation, which places the implant above the pectoralis major muscle, is associated with reduced postoperative pain and quicker recovery, largely due to the absence of muscle involvement. This technique also avoids animation deformity, enhancing the natural appearance of the breast and contributing to higher patient satisfaction. Studies like those by Franceschini et al. and Catellani et al. found that prepectoral reconstruction provided superior aesthetic outcomes and greater patient satisfaction, with patients reporting less pain and a quicker return to normal activities. Overall, the prepectoral approach appears more favorable in terms of both comfort and patient contentment post-surgery.

The various studies on transaxillary breast augmentation indicate a nuanced relationship between postoperative pain, patient satisfaction, and other outcomes. Generally, postoperative pain is reported to be minimal or well-managed across the studies, particularly when endoscopic techniques and careful tissue handling are employed. For instance, Sim HB’s study reported that 84% of patients returned to normal life within three days, highlighting minimal pain and quick recovery. Similarly, Esposito E et al. found effective pain management with no major complications, attributing this to preoperative blocks and careful surgical technique. Patient satisfaction remains high in most studies, with emphasis on the absence of visible scarring on the breast, a significant cosmetic advantage of the transaxillary approach. Studies by Philippe A. Giordano et al., Aygit AC et al., and Momeni A. et al. all reported high satisfaction rates, supported by minimal complication rates and effective aesthetic outcomes. Additionally, other findings suggest that while the transaxillary approach may have a steeper learning curve and slightly longer operative times, it offers a favorable profile in terms of scar quality and complication rates, making it a viable and often preferred option for patients concerned about visible scarring and looking for a rapid recovery.

The studies on periareolar breast augmentation consistently highlight favorable outcomes in terms of postoperative pain and patient satisfaction. Generally, patients report minimal postoperative pain with this technique, attributed to the controlled surgical environment and precise dissection allowed by the periareolar incision. For example, the study by Klinger M et al. noted modest pain levels that decreased over time, paralleling the surgical team's growing experience. This effective pain management contributes significantly to high patient satisfaction across various studies. Patient satisfaction is notably high with the periareolar approach, largely due to the subtle scarring and natural aesthetic results achieved. Moltó-García R et al. reported that 71.43% of patients rated their outcomes as very positive, while Byun IH found an average satisfaction score of 9.02 on the Likert scale, emphasizing the aesthetic appeal of minimal visible scarring. Additionally, Muhammad Humayun Mohmand and Muhammad Ahmad observed high satisfaction rates, with only a small percentage of patients experiencing temporary sensation changes or minor scarring issues. Other findings across these studies indicate that the periareolar approach is versatile and effective for a variety of breast procedures, including augmentation, mastopexy, and corrections of deformities. Complication rates are generally low, with minor issues such as scar widening and capsular contracture being rare and typically manageable. The technique's ability to provide precise surgical control and aesthetically pleasing results makes it a preferred choice for patients seeking minimal scarring and effective outcomes.

The studies on transumbilical breast augmentation (TUBA) highlight several key findings regarding postoperative pain and patient satisfaction. TUBA generally results in low postoperative pain, as indicated by studies such as Brennan WA and Haiavy J, where patients reported only requiring narcotics for a short period (48-72 hours). Similarly, Huang et al. and Won Lee et al. observed minimal postoperative discomfort and high patient satisfaction, suggesting that TUBA effectively minimizes pain compared to other augmentation techniques. Patient satisfaction with TUBA is notably high due to the technique's ability to provide a scar-free breast augmentation. Huang et al. found high satisfaction with full endoscopic monitoring, while Won Lee et al. and Won Lee and Seong Hwan Kim reported that patients were pleased with the aesthetic outcomes and minimal scarring. Notably, the technique's success is supported by Riggio et al., who reported high satisfaction with combined abdominoplasty and TUBA, enhancing body image and overall satisfaction. Other findings indicate that TUBA faces challenges such as the need for specialized training and the inability to use prefilled implants directly. Despite these limitations, the technique offers effective results with minimal complications, including low rates of infections and scar issues. Overall, TUBA is valued for its minimal scarring, effective pain management, and high patient satisfaction, making it a viable option for breast augmentation.

The findings from various studies reveal a range of complications associated with different breast augmentation techniques. Injectable materials such as paraffin, silicone, and hydrophilic polyacrylamide hydrogel historically resulted in severe complications, including skin necrosis, infection, and delayed cutaneous reactions. Modern silicone implants have improved in safety but still present risks like implant rupture, capsular contracture, and silicone migration. Textured implants have been associated with a lower incidence of capsular contracture compared to smooth implants, although long-term benefits need further investigation [54]. Autologous fat grafting, while generally safer, can still cause erythema, cysts, and abscesses. Studies on acellular dermal matrix (ADM)-assisted breast reconstructions show varied outcomes. ADM use often results in higher rates of complications like seroma, infection, and reconstructive failure compared to non-ADM procedures [59-60]. However, sterile human ADM shows a low complication rate and successful graft incorporation, indicating it may offer a better risk profile [63]. Transfeminine patients undergoing breast augmentation face higher risks for hematoma, seroma, and implant malposition compared to cisgender women [56]. Overall, while advancements have reduced some risks, careful surgical planning and patient management remain essential to mitigate these complications.

The future of breast augmentation is poised for significant advancements driven by innovations in surgical techniques, biomaterials, and patient-centered approaches. One promising avenue is the refinement of acellular dermal matrices (ADM) and their integration with autologous fat grafting, which could further reduce complications like capsular contracture while enhancing aesthetic outcomes. The development of more cohesive and durable implant materials will likely decrease rupture rates and improve long-term patient satisfaction. Additionally, non-invasive or minimally invasive techniques, such as advancements in endoscopic procedures, may provide even safer options with shorter recovery times and fewer visible scars. Personalized medicine will play a pivotal role, with techniques increasingly tailored to individual patient anatomy and preferences, potentially supported by 3D imaging and printing technologies for pre-surgical planning. Moreover, regenerative medicine and stem cell therapies could offer new possibilities in breast reconstruction and augmentation, leading to more natural and sustainable results. The focus will also shift towards enhancing patient education and shared decision-making, ensuring that individuals are fully informed of the risks and benefits of each option. As these advancements converge, breast augmentation is set to become safer, more effective, and more aligned with the evolving expectations of patients.

In summary, the choice of breast augmentation technique significantly impacts postoperative outcomes, patient satisfaction, and complication rates. Prepectoral augmentation stands out for its favorable results, offering reduced postoperative pain and enhanced aesthetic outcomes due to the absence of muscle involvement. This technique contributes to higher patient satisfaction, aligning with findings that highlight its superiority in terms of comfort and natural appearance. Transaxillary augmentation is another strong option, providing minimal scarring and effective pain management, although it requires advanced surgical skills. Its high patient satisfaction underscores its appeal for those seeking a scar-free result. Periareolar augmentation demonstrates consistent success with minimal postoperative pain and high satisfaction rates, benefiting from precise surgical control and subtle scarring. Transumbilical augmentation (TUBA) also excels with its scar-free approach, combining low pain and high satisfaction, though it faces challenges like the need for specialized training. However, complications remain a critical consideration across techniques. Historical use of injectable materials posed severe risks, while modern silicone implants and textured variants offer improved safety but still present potential issues like rupture and capsular contracture. Autologous fat grafting and acellular dermal matrix (ADM) reconstructions offer varied outcomes, with some ADM types showing higher complication rates, though advancements like sterile human ADM have shown promising results. Overall, while advancements in techniques and materials have enhanced safety and patient satisfaction, careful surgical planning and patient management are essential to mitigate risks and ensure optimal outcomes. Each technique has its advantages and limitations, necessitating a tailored approach to meet individual patient needs and preferences.

## Conclusions

Breast augmentation has evolved into a multifaceted field, offering a range of techniques tailored to meet individual patient needs. The choice of technique, whether it be prepectoral, subpectoral, transaxillary, periareolar, or transumbilical, significantly impacts postoperative outcomes, patient satisfaction, and complication rates. Prepectoral augmentation emerges as a superior method, reducing postoperative pain and enhancing aesthetic outcomes, thereby leading to higher patient satisfaction. The transaxillary and transumbilical approaches offer the unique advantage of scar-free results, which appeals to patients concerned about visible scarring, though these techniques require specialized surgical skills. The periareolar approach consistently delivers high satisfaction due to its minimal scarring and precise control, making it a versatile choice for various breast procedures. Despite these advancements, the field must continue to address the associated risks and complications, such as implant rupture, capsular contracture, and infection. The integration of acellular dermal matrices and the increasing use of autologous fat grafting present promising avenues to mitigate these risks, though they require further investigation to optimize safety and efficacy.

The future of breast augmentation lies in the personalized approach, where surgical planning is meticulously aligned with the patient’s anatomical and aesthetic goals. Continued research and innovation will be crucial in refining techniques, enhancing safety, and ultimately improving patient outcomes. As the field progresses, the emphasis must remain on balancing aesthetic aspirations with the imperative of minimizing risks, ensuring that breast augmentation continues to be a safe, effective, and satisfying choice for patients.
